# Leveraging Immunological Properties of Nucleic Acid Nanoparticles to Improve Cancer Therapy

**DOI:** 10.59566/isrnn.2025.0201b

**Published:** 2025-04

**Authors:** Tanvi S. Bolarum, Marina A. Dobrovolskaia

**Affiliations:** 1 Nanotechnology Characterization Lab., Cancer Research Technology Program, Frederick National Laboratory for Cancer Research sponsored by the National Cancer Institute. Frederick, MD 21702, USA;; 2 University of Maryland, College Park, MD 20742, USA

**Keywords:** Nucleic Acid Nanoparticles, NANPs, immunology, cytokines, cancer immunity cycle

## Abstract

Cancer is a systemic disorder resulting from altered molecular and cellular processes and dysfunctional body systems. One of the systems dysfunctional in cancer is the immune system. Tumors develop multiple mechanisms to escape immune surveillance and reprogram the immune cells to support cancer progression. The vicious circle of tumor–immune system interactions in cancer is often referred to as the cancer immunity cycle. Nucleic acid nanoparticles (NANPs) have recently emerged as a novel class of materials with a broad spectrum of therapeutic modalities and properties distinct from traditional therapeutic nucleic acids. Herein, we review the current literature regarding the recognition of NANPs by the immune system and explore how their intrinsic and controlled immunological properties can be leveraged to overcome the barriers to effective cancer immunotherapy created by the cancer immunity cycle.

## INTRODUCTION

Naturally occurring nucleic acids play an essential role in cellular function, disease, and heredity. Nucleic acids such as DNA and RNA provide the genetic instructions for all living organisms. Deoxyribonucleic acid, DNA, encodes for information, including development, function, and reproduction. Ribonucleic acid (RNA) serves multiple functions. For example, transfer RNA (tRNA) and ribosomal RNA (rRNA) are involved in the synthesis of ribosomal proteins, while messenger RNA (mRNA) carries the genetic code necessary for protein synthesis.

These nucleic acids have unique structures and functions that have shaped the field of nucleic acid therapeutics. Specifically, the ability of nucleic acids to form double helices and fold into complex 3D secondary structures is fundamental to their function of storing and transmitting genetic information over long periods of time. Harnessing these abilities, nucleic acids can provide valuable contributions to the field of nucleic acid therapeutics. During their preclinical development from the late 1980s to early 2000s, it was realized that therapeutic nucleic acids have unique properties that justify their separation into a separate class of drug products distinct from small molecules and protein therapeutics^1^. Some of these materials, such as antisense oligonucleotides, siRNA, miRNA, and mRNA, have already contributed unique mechanisms of action and demonstrated therapeutic advantages.

Small interfering RNA (siRNA) are double-stranded oligonucleotides that consist of sense and antisense strands, which work together to silence gene expression through mRNA degradation^2^. siRNAs have provided a novel path into treatments for hepatic diseases^2^. For example, the first siRNA drug, ONPATTRO, was approved by the US Food and Drug Administration (FDA) in 2018 for treating polyneuropathy in hereditary transthyretin amyloidosis using an LNP formulation^2^. Subsequently, five more liver-targeted siRNA drug products - GIVLAARI, OXLUMO, LEQVIO, AMVUTTRA, and RIVFLOZA - utilizing an N-acetylgalactosamine (GalNAc) conjugate for specific liver localization received regulatory approval for clinical use^2^. MicroRNAs (miRNAs) are small, non-coding RNA molecules that regulate post-transcriptional gene expression. They continue to be studied as an approach for regulating gene expression in various diseases, particularly cancer, metabolic disorders, and fibrosis. Several lipid-nanoparticle-based formulations that deliver mRNA-encoded SARS-CoV-2 antigen – Comirnaty and Spikevax - have been approved by the FDA for the prevention of COVID-19 and have paved the way for developing LNP-mRNA technology for other therapeutic applications^3^.

Antisense oligonucleotides (ASN) are single-stranded DNA molecules that hybridize with RNA via the classic Watson-Crick base-pair mechanism and reduce, restore, or modify protein expression by altering RNA function^4^. A couple of notable achievements include regulatory approval of mipomersen (also known under the brand name Kynamro^®^) for hypercholesterolemia in 2013, inotersen (Tegsedi^®^) for hereditary transthyretin amyloidosis^3^ in 2018 and tofersen (Qalsody^®^)for amyotrophic lateral sclerosis in 2023^2^.

Splice-switching oligonucleotides (SSOs), which modulate pre-mRNA splicing to restore functional protein expression, have also shown remarkable success. Particularly with nusinersen (Spinraza^®^) for spinal muscular atrophy (SMA), SSOs have significantly impacted SMA disease progression^2^. Remarkably, the n-of-1 SSO, Milasen, demonstrated the potential for rapid, personalized genetic treatments^2^. Other development milestones, achievements, and delivery challenges of traditional therapeutic nucleic acids have been reviewed in detail elsewhere^5^.

Following these traditional therapeutic nucleic acid discoveries, nucleic acid nanoparticles (NANPs) have emerged as a promising approach to enhance drug delivery and therapeutic efficacy. Nucleic acid-based nanomaterials enable exact molecular recognition, mitigated self-assembly, and versatile biofunctionalization. NANPs exhibit a programmable nature, compatibility with biological systems, and consistent performance, and they work very precisely with therapeutic nucleic acids, optimizing stability and intracellular delivery^6^. NANP technology provides a platform for the construction of dynamic nanostructures that can respond to environmental stimuli for precise therapeutic interventions^6^. The size, shape, stoichiometry, module density, surface chemistry, composition, hydrophobicity, and elasticity of nucleic acid nanoparticles contribute to their biological performance^7^. Assembling traditional oligonucleotides into nanostructures with varying sizes, shapes, and chemical modifications increases their stability under strenuous conditions, such as thermal, radiation, chemical, and enzymatic degradation, and creates unique functionalities not attainable by traditional nucleic acids^8^. Not surprisingly, NANPs’ comparison to traditional therapeutic nucleic acids, small-molecule drugs, and biologics using the criteria proposed by the Drug Information Association Oligonucleotide Safety Working Group suggested that NANPs qualify for a separate group of therapeutic products^9^.

NANPs’ application in oncology, along with opportunities, achievements, and challenges with their translation from the bench to the clinic, have been extensively discussed elsewhere^7, 8, 10^. Herein, we focus on the potential of this technology to improve cancer therapy by leveraging the inherent immunological properties of NANPs to overcome barriers created by the so-called cancer immunity cycle. Due to the breadth of NANP technologies, this review specifically focuses on DNA and RNA nanoparticles.

## THE IMMUNE SYSTEM AND CANCER

### Cancer Immunity Cycle

**Fig. 1.**
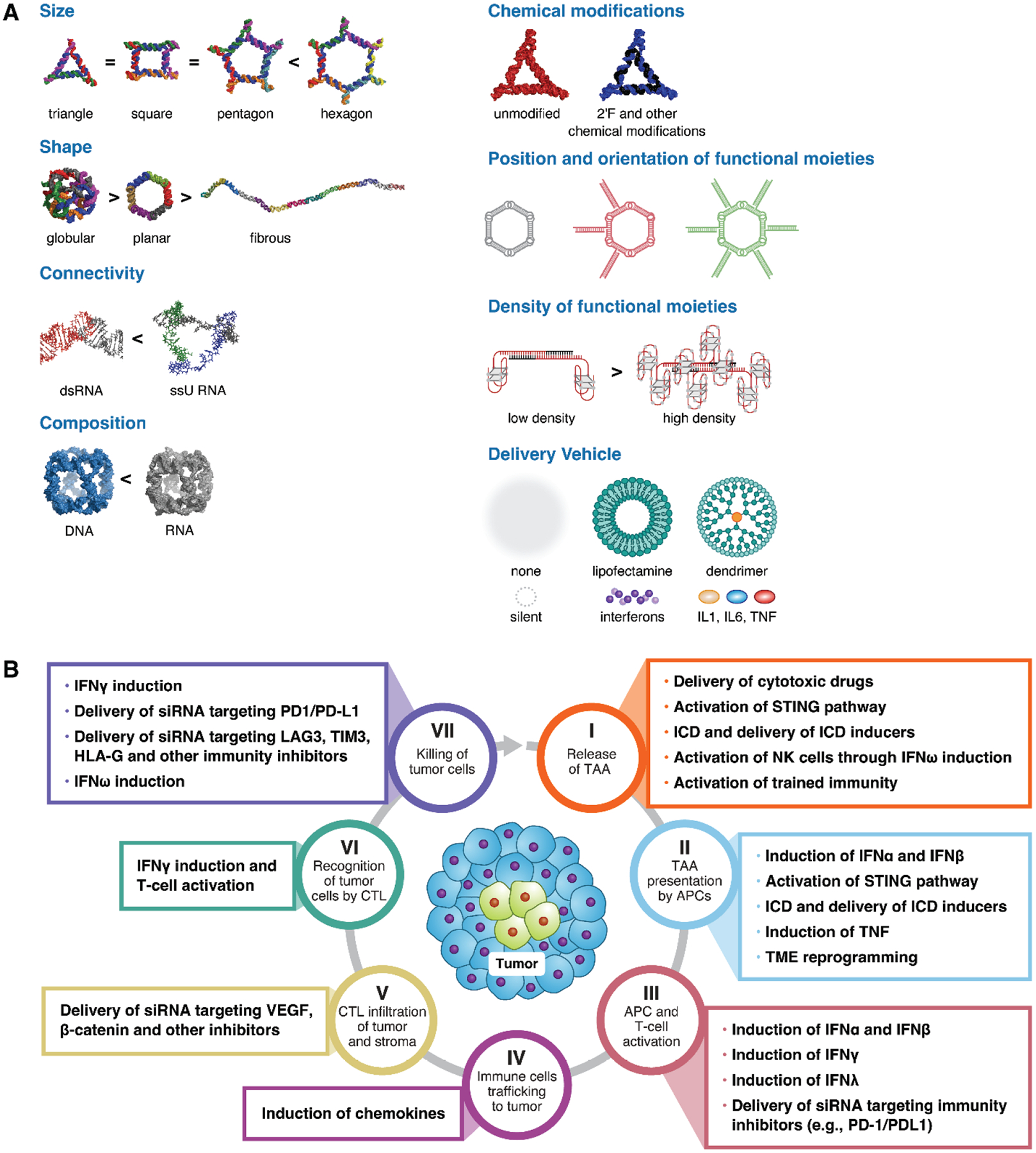
Immunological properties of NANP and their potential application to combat cancer immunity cycle. (A) Key structure-activity relationships currently known to underlie the inherent immunological properties of NANPs are summarized. The information is summarized based on references^102–104, 107, 109, 110^ (B) The seven key stages of the cancer immunity cycle are shown as conceptualized elsewhere^12^. Currently known properties of NANPs that may have either direct, through inherent immunological properties, or indirect, through the delivery of drugs and gene therapies, effects on various stages of the cancer immunity cycle to improve cancer immunotherapy are summarized in rectangular text boxes, which are color-coded to match the corresponding step in the cancer immunity cycle. TNF – tumor necrosis factor; IFN – interferon; IL- interleukin; ICD – immunogenic cell death; TAA – tumor-associated antigens; APC – antigen-presenting cells; CTL – cytotoxic T-lymphocytes; HLA – human lymphocyte antigens; PD – programmed death; PD-L – programmed death ligand; LAG - lymphocyte activation gene; TIM - T-cell immunoglobulin and mucin domain; STING - stimulator of interferon genes; VEGF – vascular endothelial growth factor; ds – double-stranded; ss – single-stranded; si – small inhibitory; NK – natural killer; TME – tumor microenvironment.

The cancer-immunity cycle concept, initially proposed by Chen and Mellman^11^, describes the key steps of how the immune system recognizes and destroys cancer cells. The cycle involves the following steps: 1) release of cancer cell antigens, 2) cancer antigen presentation, 3) priming and activation of T cells, 4) trafficking of T cells to tumors, 5) infiltration of T cells into tumors, and the stroma, 6) recognition of cancer cells by T cells, and finally, 7) killing of cancer cells^11, 12^. These steps, along with other details, are presented in Figure 1. In step one, tumor cells die via apoptosis or necrosis, releasing tumor-associated antigens (TAAs). Challenges at this step include the poor immunogenicity of some tumors, hindering antigen release, and how some tumors can overcome cell death by overexpressing anti-apoptotic proteins^11^. In step two, antigen-presenting cells (APCs) process tumor antigens and present them on major histocompatibility complex (MHC) molecules. However, tumors can secrete immunosuppressive factors that impair dendritic cell maturation, and some tumors may even downregulate MHC expression^11^. Step three involves the priming and activation of T cells in lymphoid organs after they recognize antigens presented by dendritic cells. At this step, tumors may express immune checkpoint molecules that inhibit T-cell activation^11^. Step four involves activated T cells traveling through the bloodstream to the tumor site. At this step, tumors downregulate the chemokines needed for T-cell infiltration^11^. During step five, T cells puncture the tumor stroma and reach the cancer cells. Here, metabolic competition limits T-cell function, and the tumor microenvironment (TME) contains immunosuppressive cells^11^. Step six involves T cells recognizing tumor cells through T-cell receptors (TCRs) that are specific to TAAs displayed on major histocompatibility complex (MHC) molecules. An issue that arises at this step includes tumors downregulating or mutating MHC molecules, which end up reducing tumor visibility to T cells^11^. The final step of the cycle occurs when cytotoxic T lymphocytes (CTLs) and natural killer (NK) cells induce apoptosis in tumor cells. At this stage, tumors may develop resistance by upregulating anti-apoptotic proteins^11^.

### Mechanisms underlying tumor immunosuppressive environment

The immunosuppressive environment created by complex interactions between cancer cells, surrounding stroma, and immune cells inhibits the normal anti-tumor immune response and promotes tumor development. The breadth of mechanisms underlying the immunosuppressive tumor microenvironment poses challenges to effective immunotherapy. Biochemical mechanisms that create barriers to effective immunotherapy include dysfunction of the antigen-presenting machinery, inhibitory ligands that suppress T-cell function, and immunosuppressive cytokines and mediators. Cellular mechanisms supporting cancer immune escape are the dysregulated function of dendritic cells, immunosuppressive cell populations, rapid neovascularization, and angiogenesis.

Alterations in MHC-I expression and the antigen-processing and presentation machinery allow tumors to evade proper recognition and lysis by cytotoxic T cells, reducing their sensitivity to killing by NK cells^13–18^. Tumors can selectively inhibit T-cell function through both contact-dependent and independent mechanisms, as well as T-cell exhaustion, by upregulating PD-L1 expression on tumor cells and PD-1 and CTLA-4 expression on T cells^19–21^. Moreover, tumors can directly kill T-cells through the induction of the Fas/FasL apoptotic pathway, either by expressing FasL on their own surface or by secreting FasL-bearing exosomes^22–24^. Tumors also disrupt dendritic cell (DC) trafficking and function by inducing DC apoptosis, suppressing maturation and differentiation, and affecting the efficiency of antigen processing and presentation^25–27^.

Tumor-associated M2 macrophages and CD4+ CD25+FoxP3+ regulatory T-cells (Tregs) suppress anti-tumor immune responses through secretion of immunosuppressive cytokines IL-10 and TGFβ, and presentation of CTLA4 on T cells and PD-L1 on macrophages^28–33^. Tumors also induce the dysfunction of other immune cells, including B-lymphocytes and neutrophils^34^. Tumors’ rapid neovascularization and angiogenesis contribute to the overproduction of vascular endothelial growth factor (VEGF) that affects DC function by upregulating PD-L1 expression and inhibiting DC maturation and antigen presentation^35^.

Hypoxia, a condition intrinsic to tumor rapid growth and vascularization, promotes VEGF secretion by tumor cells and M2 macrophages and TGFβ production by M2 macrophages, which collectively upregulate PD-L1 expression on cancer cells and CTLA4 ligand CD86 on DCs, and inhibit tumor cell recognition through costimulatory receptor NKG2D on NK and T cells^36^. Compression of blood and lymphatic vessels by tumor mass results in elevated intratumoral interstitial pressure, which, in addition to promoting hypoxia, results in a buildup of metabolic waste products in the tumor, leakage of nutrients, drugs, and cells out of the tumor, and prevents effective delivery to and retention in the tumor of therapeutic agents^37, 38^.

Low expression of critical adhesion molecules such as VCAM1, ICAM1, ICAM2, and E-selectin on endothelial cells comprising tumor vasculature inhibit effective traffic of immune cells from the peripheral circulation to the tumor site^39^. In contrast, the overexpression of receptors involved in the transmigration of Tregs and other immunosuppressive cells on the surface of tumor endothelium, along with upregulation of inhibitory proteins such as TGFβ, FasL, and PD-L1, create conditions preventing the effective anti-tumor immune response^40–42^.

### Traditional immunotherapy approaches

To overcome these barriers, a variety of recombinant cytokines, antibodies targeting inhibitory proteins affecting T-cell function, vaccines, and cell and gene therapies have been investigated. Some passed regulatory reviews and received FDA approval for clinical use (Table 1). Recombinant cytokine IL-2, for example, was among the first biologics approved for cancer therapy. Serving as an essential growth factor for T-lymphocytes, it activates and expands cytotoxic T cells and NK cells^43^ leading to tumor regression in ~ 15% of patients^44, 45^. Another recombinant cytokine, IFN-α, approved for therapy of AIDS-related Kaposi’s sarcoma, melanoma after surgical resection, hairy cell leukemia, and renal cell carcinoma in combination with Avastin^46–48^, directly inhibits the proliferation and function of tumor cells, increases the expression of tumor-associated antigens on tumors, activates dendritic and NK cells, and creates conditions favoring anti-tumor immunity by stimulating cross-priming of cytotoxic T cells by DCs^49–51^. Due to their immunostimulatory mechanism of action and pleiotropic nature, recombinant cytokines overcome tumor-mediated immunosuppression through various mechanisms.

Tumor-infiltrating lymphocytes (TILs) were successfully employed for adoptive immunotherapy with overall response rates of 50% or higher^52, 53^. In this approach, TILs extracted from tumors are expanded *ex vivo* using IL-2 before infusion into the same patients^54^. Lymphodepletive chemotherapy and radiation may be applied before TIL infusion to remove endogenous lymphocytes, such as Tregs, which may prevent TILs from completing their intended function.

Chimeric antigen receptor (CAR) T cells are genetically modified to express antibody-derived targeting ligands fused to T cell receptor (TCR) and additional costimulatory ligands^55^. After the initial success of targeting CD19 on B-cell refractory lymphomas^55^, NY-ESO-1-targeting on multiple myeloma^56^ and synovial cell carcinoma^57^, various generations of CAR-T therapies have been developed and paved the way for other chimeric antigen receptor cells such as CAR-NK, CAR-macrophages, and CAR-Tregs^58^.

Immune checkpoint inhibitors, including anti-CTLA-4, anti-PD1, and PD-L1, represent another immunotherapy group that targets immunosuppressive tumor environments. Therapeutic CTLA-4 blockade prolongs T-cell activation^59^. The PD-1 and PD-L1 blockade overcomes the exhaustion of T cells triggered by PD-L1 ligation^60^. The median response duration achieved with these inhibitors is 1–3 years^61^. The combination of these checkpoint inhibitors with a range of other strategies, including cytotoxic oncology drugs, adoptive cell therapy, and cancer vaccines, is being actively investigated for additional benefits^62–69^

Sipuleucel-T is the first cancer vaccine approved for prostate cancer; it is comprised of a cancer patient’s DCs treated *ex vivo* with an antigen-cytokine fusion protein^70^. Sipuleucel-T works by increasing the number of antigen-presenting cells that are educated ex vivo into tumors. Another cancer vaccine approach - Imlygic (T-VEC) – is based on the oncolytic virus that lyses tumor cells *in vivo* to release tumor-associated antigens and induce tumor-specific immunity with the aid of DC and monocyte activating GM-CSF expressed from a co-delivered gene encoding this cytokine^71^.

While more drug candidates to improve the outcomes of cancer immunotherapy have been developed and advanced to clinical trials, many challenges still remain, including but not limited to the high heterogeneity of response between individual patients, lack of predictive biomarkers for selecting responders, tumor heterogeneity, tumor resistance, and high overall costs^15, 72^.

### Benefits of nanotechnology

Nanotechnology offers several advantages to cancer immunotherapy (Table 2). Among them are the improved drug delivery to the tumor via enhanced permeability and retention (EPR) and enhanced transcytosis and retention (ETR) mechanisms, cell- and tissue-targeted delivery, and intrinsic immunostimulatory, immunomodulatory, and hemomodulating properties of nanocarriers^73, 74^. In the clinic, nanotechnology formulations Doxil, Abraxane, and Onivyde are used to deliver cytotoxic drugs while reducing systemic toxicities and maintaining the therapeutic efficacy of the active pharmaceutical ingredients doxorubicin, paclitaxel, and irinotecan, respectively^75–77^ Combination immunotherapies delivered or co-delivered using nanoparticles include immune-activating or immunosuppressive therapies that complement traditional chemotherapy, radiation therapy, or vaccines.

Nanoparticles enhance the abscopal effect, a unique phenomenon in which the local elimination of cancer cells induces a systemic anti-tumor immune response, thereby clearing distant tumors. Nanoparticles can be designed to capture tumor-associated antigens released during radiation therapy, transport these antigens to antigen-presenting cells, activate the antigen-presenting cells, trigger the secretion of cytokines and other proinflammatory factors to make tumors “immunologically hot,” and reprogram the tumor microenvironment to increase tumor susceptibility to immune attack^78–88^. Nanoparticle physicochemical properties, such as size, have been shown to improve the lymphatic drainage of nanoparticle-bound antigens. Smaller particles, with a 30–50 nm hydrodynamic size, travel quickly through lymphatic vessels to lymph nodes after subcutaneous (s.c.) administration, whereas larger particles with size ≥ 100 nm remain at the injection site^89–91^. CYT-6091, a PEGylated gold nanoparticle-based formulation of tumor necrosis factor-alpha, besides the improved safety vs. free cytokine, reduced interstitial intratumoral pressure, thereby improving the delivery of anticancer drugs to tumor^92^.

## IMMUNOLOGICAL PROPERTIES OF NANPS

### Hemocompatibility

Hemocompatibility is commonly assessed by analyzing a test substance’s effects on erythrocyte integrity, platelets, plasma coagulation, and complement activation^93^. While initially developed for analyzing medical devices, these tests were adopted for nanoparticle characterization^73, 94, 95^ and, due to good *in vitro*-*in vivo* correlation^96^, found broad use in the preclinical development of nanomedicines^97,98^. Fibrous NANPs did not activate the complement system *in vitro* and resulted in weak prolongation of activated partial thromboplastin time without affecting prothrombin and thrombin times^99^. Three-way junction 3WJ RNA nanoparticles – both the scaffold and scaffold functionalized with siRNA - were not hemolytic, did not induce platelet aggregation, did not affect collagen-induced platelet aggregation, and did not result in the prolongation of plasma coagulation time^100^. 3WJ scaffold resulted in a weak activation of the complement system^100^. Interestingly, the attachment of siRNA to the 3WJ nanoparticle scaffold reduced complement activation^100^. While extensive studies on the hemocompatibility of NANPs are not available, published studies collectively suggest a high degree of hemocompatibility of NANPs at submicromolar concentrations that are also commonly used for the therapeutic applications of these materials.

### Inflammatory responses and cytokines

Due to their macromolecular nature, NANPs are immunoquiescent at submicromolar concentrations, which contrasts them from traditional immunostimulatory nucleic acids such as CpG oligonucleotides. It has also been shown that the immunostimulatory properties of NANPs in the absence of a complexation agent could be enhanced by the addition of special immunostimulatory single-stranded RNA sequences^101^. When forced into cells using transfection reagents, NANPs induce a pro-inflammatory response. The magnitude and breadth of this proinflammatory response depends on the route of cellular entry, which in turn is determined by the type of the delivery vehicle. For example, when Lipofectamine 2000 (L2K) is used to complex NANPs before the addition to human peripheral blood mononuclear cells (PBMC), a very specific interferon response, encompassing type I and type III interferons (IFNs), is induced with a sharp structure-activity relationship (SAR) (Figure 1A). Specifically, globular NANPs, such as RNA cubes, are more potent at inducing type I and III IFN response than DNA cubes of the same size and molecular weight^102^. Among RNA-based NANPs, globular structures such as RNA cubes are more potent than planar structures such as RNA rings, which in turn are more potent than fibrous structures such as RNA fibers with comparable molecular weight^102^. The IFN response to L2K-delivered RNA rings varies depending on the number and position of functional RNA moieties attached to the ring scaffold^103^. Likewise, the IFN response to L2K-delivered RNA/DNA fibers could be further controlled by adjusting the density of aptamers attached to the fiber scaffold – the higher the density, the lower the IFN response^104^. Another interesting SAR aspect of L2K-delivered NANPs is that, while nanoparticle size matters, the apparent difference in IFN response is observed only with the largest particles, such as hexagons. In contrast, no clear difference is observed between triangles, squares, and pentagons. The presence of uracils at the corners of RNA cubes and RNA rings is essential for the induction of IFN response, whereas sequence complementarity (sense vs. antisense) does not matter^102^. Likewise, in the human microglia cell line, L2K-delivered NANPs induced an interferon response depending on their molecular weight, melting temperature, and half-life^105^.

While monocytes, myeloid DCs, and monocyte-derived DCs contribute to the NANP-mediated IFN response, pDCs were found to be the primary responders among the cells present in the complex PBMC cultures^102^. Interestingly, similarly to traditional CpG oligonucleotides, such as ODN2216, the L2K-complexed NANPs induced IFN response after being processed via phagolysosomal pathway^102^. However, in contrast to ODN2216, NANPs intracellular distribution and trafficking were only possible after L2K complexation^102^. The ODN2216 is a CpG oligo with a phosphodiester backbone in the middle and a phosphorothioate backbone at the flanks, originally identified as a selective activator of pDCs; it does not require a transfection reagent to activate pDCs.

Another contrasting property is the fact that PBMC internalize L2K-NANP complexes via scavenger receptor and that TLR7, a pattern recognition receptor (PRR) for single-stranded RNA, instead of TLR3, a PRR for double-stranded RNA, is involved in the IFN response to RNA-cubes^106^. While the exact mechanism underlying these differences is unknown, one can hypothesize that the slower decomposition of macromolecular NANPs within the endosomal compartment may release single-stranded RNA fragments that react with and activate TLR7. Another cytosolic PRR – cGAS – was shown to mount a pro-inflammatory response to RNA/DNA fibers in THP-I reporter cells^104^, demonstrating further complexity and breadth of opportunities to control NANPs-mediated inflammatory responses. RIG-I - a PRR responsible for the recognition of 5’- triphosphorylated RNA - was shown to drive cellular IFN response to RNA-based NANPs, and, interestingly, contribute to the intracellular recognition of DNA-based NANPs^107^. The mechanism underlying the unexpected recognition of DNA-based NANPs was attributed to DNA-dependent RNA polymerase III, which transcribes AT-rich regions of double-stranded DNA. IFN response to L2K-delivered DNA-NANPs was reduced in microglial cells exposed to siRNA, inhibiting the expression of the catalytic subunit of RNA polymerase III^107^.

Another remarkable immunological property of NANPs is that the breadth, depth, and quality of the pro-inflammatory response to these materials change depending on the type of delivery vehicle employed to achieve their internalization by immune cells^108, 109^. The SAR properties discussed above apply to NANPs delivered using a lipid-polymer-based transfection reagent L2K. When other lipofectamines such as lipofectamine messenger max (LMM) are used, the cell populations taking up NANPs and pro-inflammatory properties remain the same, but the degree of the uptake by the lymphocyte subpopulations becomes higher than in L2K-delivered group^109^. Interestingly, unlike IFN response to LMM-delivered RNA cubes and rings, that to LMM-delivered RNA fibers was lower than to their L2K-delivered counterpart^109^; the difference possibly results from the lipofectamine composition in that LMM, unlike L2K, is optimized to deliver fibrous nucleic acids such as mRNA.

When the delivery vehicle is changed to another class of materials, such as cationic PAMAM dendrimers, the spectrum of the PBMC-produced cytokines changes dramatically – instead of the IFN response seen with L2K-delivered NANPs, proinflammatory stress and danger biomarkers – TNF, IL-1α, IL-1β, and IL-6 – are observed with amine-terminated PAMAM dendrimers-delivered NANPs^108^. Similar changes were also reported in microglial cells in that change in the delivery vehicle from L2K to cationic amphiphilic co-polymer, poly (lactide-coglycolide)-graft-polyethylenimine (PgP) completely abrogated type I IFN response^110^. Likewise, when NANPs delivery vehicle was changed to DOTAP – a cationic lipid – the NANPs’ uptake by immune cells became lower than that observed with L2K-mediated delivery of the same NANPs^107^. The key immunological SARs are summarized in Figure 1A. While the data available in the current literature utilize research-grade transfection reagents such as lipofectamine, we envision that future research will involve other delivery vehicles optimized for clinical use. The current data will guide the selection, design, and optimization of such NANP carriers to achieve the desired immunological properties. Rapidly evolving computational approaches will aid this process.

### Computational approaches

The breadth of structural designs and well-defined structure-activity relationships opened possibilities to leverage experimental findings of NANP’s immunological properties in computational machine learning studies. Molecular dynamics simulation approaches were successfully used to assess the dynamics and flexibility of NANPs, as well as predict their interaction with several PRRs involved in their immunological recognition^6, 99, 104, 111–116^.

Combining the NANP molecular weight and melting temperature with type I IFN secretion data for machine learning using the random forest approach led to the development of a quantitative structure-activity relationship model (QSAR)^105^. Expanding this work further allowed researchers to develop several modifications of the transformer algorithm, including transformer M2, that very accurately predict type I and type III interferon responses to L2K-delivered NANPs based on the sequences of oligonucleotides used to compose them^117^. This work led to the development of the artificial immune cell platform AI-Cell, which is currently available to the public on the National Center for Advancing Translational Sciences website (https://aicell.ncats.io/).

While molecular dynamics simulation and AI-Cell cannot replace immunological studies of NANPs, they provide tremendous help with research prioritization. In a combined computational biology and immunology approach, one can first utilize existing computational platforms to select NANPs with desired immunological properties and then conduct specialized immune function tests using the pre-selected NANPs, thereby reducing the time and costs associated with the preclinical characterization of NANP-based drug products.

### Therapeutic potential with demonstrated proof-of-concept

RNA-DNA fibers designed to release NFkB-decoy oligonucleotides efficiently reduce the production of NFkB-dependent cytokines such as TNF and IL1 induced by bacterial LPS in human PBMC cultures^118^, a property that may offer a therapeutic benefit in LPS-mediated inflammation-associated conditions like sepsis. The production of type I IFN by osteoclasts and osteoblasts in response to L2K-delivered NANPs benefits bone infection therapy by reducing *S. aureus’* burden in bones^119^. Their immunoquiescent nature and ability to add functional moieties allowed researchers to explore fibrous NANPs for the delivery of thrombin aptamers to control blood coagulation *in vitro* and *in vivo*^99, 120, 121^. Unlike traditional aptamers and standard-of-care blood thinning agents such as heparin, which may lead to excessive bleeding, the anticoagulant properties of NANPs-formulated thrombin aptamers could be controlled via complementary moieties through the so-called “kill-switch” mechanism^99^. Though not based on their immunological properties, NANPs were also shown to benefit cancer therapy by delivering anticancer drugs (e.g., paclitaxel, camptothecin, SN38) and therapeutic nucleic acids such as aptamers and siRNA that inhibit tumor cell proliferation (e.g., by targeting survivin, KRas, and EGFR pathways)^100, 122–126^.

The intrinsic properties of nucleic acids, controlled SAR of NANPs, and breadth of mechanisms to control their immunological recognition—from structural design to delivery inside the cells—open many potential applications for these materials in disease diagnosis, prevention, and therapy. Below, we will focus on one such area: cancer immunotherapy.

## PERSPECTIVE OF USING NANPS FOR CANCER IMMUNOTHERAPY

The inherent immunostimulatory properties that depend on NANP’s size, shape, composition, and connectivity can be further controlled by chemical modifications, position, orientation, and density of functional moieties, as well as the type of delivery vehicle (Figure 1A), making NANPs a versatile tool for controlled immunostimulation. These properties open up numerous possibilities for utilizing NANPs as vaccine adjuvants, *in situ* cancer vaccination agents, and immunotherapeutics. When combined with design principles that allow the use of NANPs for the delivery of cytotoxic oncology drugs, immunogenic cell death inducers, regulatory nucleic acids, and oligonucleotide-based targeting functional moieties, these properties offer broad potential to overcome the barriers created by the cancer immunity cycle (Figure 1B).

The inherent ability of NANPs to induce interferon responses when delivered using a lipid-based vehicle may overcome barriers created by dysfunctional antigen presentation machinery because IFNω can activate natural killer (NK) cells involved in direct killing of tumor cells, IFNα and IFNβ activate dendritic cells involved in antigen processing and presentation whereas IFNλ and IFNγ activate both APCs and T-cells^127^. Therefore, only through the induction of beneficial interferon responses can lipid-based carrier-delivered NANPs change the activation status of immune cells and contribute to overcoming five of seven steps^12^ of the cancer immunity cycle: I. Release of tumor-associated antigens, II. TAA presentation by APCs, III. APC and T-cell activation, VI. Recognition of tumor cells by CTLs, and VII. Killing of tumor cells. NANPs designed to deliver siRNAs against cancer immunity-inhibitory machinery – e.g., PD-1 and CTLA4 on T-cells, PD-L1 on cancer cells and APCs, VEGF on tumor vasculature, LAG, TIM3, HLA-G, TGFβ on tumor cells – may aid in overcoming barriers created by four steps of the cancer immunity cycle: III. APC and T-cell activation, V. CTL infiltration of tumors and stroma, and VII. Killing of tumor cells. The induction of chemokines and proinflammatory cytokines such as TNF, IL1α, and IL-1β by NANPs complexed with polymer-based and cationic delivery vehicles may help overcome the barriers created by step II. TAA presentation by APC, and step IV. Immune cells trafficking to tumors. The delivery of cytotoxic oncology drugs by NANPs and their intrinsic ability to activate the STING pathway may also aid with improving TAA release by tumor cells.

While most established immunotherapies target adaptive immunity, the induction of trained immunity – also known as innate immune memory or immunomodulation – recently gained the attention of cancer researchers as an additional tool to fight cancer cells^128^. In the trained immunity approach, cancer regression is achieved by optimizing the tumor microenvironment through epigenetic changes and metabolic reprogramming of innate immune cells, which, in turn, create more permissive conditions for T-cells to kill cancer cells more effectively. While BCG and fungal glucans are commonly used as inducers of trained immunity, other agents may have similar properties. The abilities of NANPs to induce cytokine, interferon, and chemokine responses, along with their recognition and activation of the STING and RIG-I pathways, warrant further investigation into the epigenetic and metabolic reprogramming of innate immune cells to more thoroughly explore the properties of these materials as inducers of trained immunity.

The ability of NANPs to induce type I and type III interferon responses suggests that the immune system recognized these materials as viruses. Therefore, a strategy recently described for plant viruses applied for cancer *in situ* vaccination^129–134^ may also apply to NANPs.

A similar technology – DNA origami – was recently demonstrated to enhance the antibody responses by multivalent presentation of antigens organized on the origami surface^135^. This strategy could be adapted to other NANPs, such as DNA/RNA nanoparticles discussed in this perspective. This offers additional possibilities for improving current immunotherapy approaches by using NANPs.

## CONCLUSION AND FUTURE DIRECTIONS

Successful immunotherapy will likely involve multiple approaches simultaneously targeting various elements of innate, adaptive, and trained immunity. NANPs, due to their versatility and programmable design principles, have the potential to become such therapies. Moreover, their inherent properties will likely benefit existing approaches by combining cytotoxic drugs and checkpoint inhibitor blockades. Future research in this area may require additional genomics, proteomics, and transcriptomics analyses to fully understand NANP’s potential for inducing trained immunity, *in situ* vaccination, and cancer vaccines, as well as its application in both stand-alone and combination therapies with current standard-of-care immunotherapies. Combinatory approaches appear promising as next steps, as they help compensate for potential limitations of NANPs and the known limitations of the standard-of-care.

## Figures and Tables

**Table 1.** Biochemical and cellular mechanisms that create barriers to effective cancer immunotherapy and current therapeutic approaches to overcome them. The table summarizes mechanisms underlying the immunosuppressive tumor microenvironment, examples of barriers they create, and current drugs approved by the FDA that help overcome these barriers. Some drugs target more than one mechanism.

Mechanisms	Examples	Current FDA-approved Drugs
Antigen-presenting machinery dysfunction	Loss or downregulation of MHC-I Downregulation of proteasome subunits needed for peptide generation Alterations in proteins involved in antigen transport to and processing in the endoplasmic reticulum	Recombinant IFNα2b (e.g., Intron and PEG-Intron) DC-vaccine (e.g., sipuleucel-T (Provenge))
T-cell anergy	PD-1 expression on T-cells PD-L1 expression on cancer cells CTLA4 expression on T-cells FasL expression on tumor cells Tumor-derived FasL-bearing exosomes	Antibody inhibiting CTLA4 (e.g., Ipilimumab (Yervoy)) Antibody inhibiting PD-1 (e.g., Keytruda (pembrolizumab)) Antibody inhibiting PD-L1 (e.g., Atezolizumab (Tecentriq))
Immunosuppressive cytokines and mediators	TGF-β expression by tumors IL-10 expression by tumor cells and tumor-resident T-cells and macrophages Aberrant chemokine production IDO-produced tryptophan metabolites Prostaglandin E2	Recombinant IL-2 (e.g., aldesleukin (Proleukin)) Recombinant IFNα2b (e.g., Intron and PEG-intron)
DC function dysregulation	Poorly differentiated DC Immature DC Tumor-driven DC apoptosis	Recombinant type I IFN therapy (e.g., Intron and PEG-intron) DC-vaccine (e.g., sipuleucel-T (Provenge))
Immunosuppressive cell populations	T-regs Immunosuppressive pDC MDSC	T-cell gene therapy (e.g., afamitresgene autoleucel (Tecelra)) DC-vaccine (e.g., sipuleucel-T (Provenge))
Rapid neovascularization and angiogenesis	Elevated intratumoral interstitial fluid pressure Hypoxia VEGF and tumor endothelium-driven immune tolerance	Intratumoral therapy (e.g., Talimogene laherparepvec (T-VEC)) Drugs targeting hypoxia (e.g., belzutifan (Welireg)) Drugs targeting VEGF and VEGF-mediated pathways (e.g., Bevacizumab (Avastin); Sunitinib malate (Sutent); Sorafenib (Nexavar); Pazopanib (Votrient); Ramucirumab (Cyramza); Brolucizumab; Fruquintinib (Fruzaqla)

IDO, indoleamine 2,3-dioxygenase; PD, programmed cell death receptor; PD-L, programmed cell death ligand; CTLA4, cytotoxic T-lymphocyte antigen-4; IL, interleukin; TGF, transforming growth factor; MHC, major histocompatibility complex; T-regs, regulatory T-cells; pDC, plasmacytoid dendritic cells; MDSC, myeloid-derived suppressor cells; VEGF, vascular endothelial growth factor. Information in the table is summarized based on references^136, 137^

**Table 2.** Application of nanotechnology to overcome common barriers to effective cancer immunotherapy. The table summarizes barriers impeding successful immunotherapy and shows some examples of nanotechnology concepts investigated to overcome these barriers. The table is prepared based on references^74, 88, 89, 92, 136, 138–152^.

Barriers	Nanotechnology-enabled approaches	Examples
Dysfunction of antigen-presenting cells	Co-delivery of TAAs Capture and concentration of TAAs Improved lymphatic delivery Local and systemic APC activation *in situ* vaccination Induction of immunogenic cell death Induction of abscopal effects Local hyperthermia induction	PLGA-AC-NPs Multiple platforms with sub 100nm size Multifunctional lipid bilayer-IDO nanoparticles CPMV SPIO SivaRods Aurosomes
Inhibited trafficking of immune cells to the tumor site	Enhance vascular permeability	CYT-6091
Anergy of immune cells	Inhibition of immune checkpoint blockade Delivery and co-delivery of immunostimulatory agents Metabolic reprogramming of TME	Multiple nanoparticle platforms to deliver IL-2, TGFβi, CpG + anti-CD40, IL-2 + anti-CD137 CPMV in situ vaccination
Altered transport and retention of drugs in the tumor	Tumor-specific accumulation of NPs through EPR and ETR effects Guidance of nanoparticles to the tumor site by external magnetic field Disruption of neovasculature	Nanoalbumin-bound drugs RGD-targeted silicasomes 10 nm GNP Hyaluronic acid functionalized NP CoFe_2_O_4_@BaTiO_3_-PTX nanostructures CYT-6091

TAA, tumor-associated antigens; CYT, CytImmune Sciences; CPMV, cowpea mosaic virus; EPR, enhanced permeability and retention; ETR, enhanced transcytosis and retention; PLGA, poly(lactic-co-glycolic acid); PTX, paclitaxel; IDO, indoleamine 2,3-dioxygenase; SPIO, superparamagnetic iron oxide nanoparticles; GNP, gold nanoparticles
